# The Influence of Concentration and Type of Salts on the Behaviour of Linear Actuators Based on PVA Hydrogel Activated by AC Power

**DOI:** 10.3390/gels11070484

**Published:** 2025-06-23

**Authors:** Aleksey Maksimkin, Mikhail Zadorozhnyy, Kseniia V. Filippova, Lidiia D. Iudina, Dmitry V. Telyshev, Tarek Dayyoub

**Affiliations:** 1 Institute for Bionic Technologies and Engineering, I.M. Sechenov First Moscow State Medical University (Sechenov University), Bolshaya Pirogovskaya Street 2-4, 119991 Moscow, Russia; aleksey_maksimkin@mail.ru (A.M.); zadorozhnyy.m.yu@mail.ru (M.Z.); kseniafilippova84013@gmail.com (K.V.F.); lida.judina0345@gmail.com (L.D.I.); telyshev@bms.zone (D.V.T.); 2 Department of Physical Chemistry, National University of Science and Technology “MISIS”, 119049 Moscow, Russia; 3 Institute of Biomedical Systems, National Research University of Electronic Technology, 124498 Moscow, Russia

**Keywords:** polyvinyl alcohol, sodium chloride, lithium chloride, hydrogel, actuator, AC voltage, artificial muscles, soft robotics

## Abstract

The creation of quick-reacting electrically conductive polymers for use as actuators driven by low electrical currents is now seen as an important issue. Enhancing the electrical conductivity of hydrogels through the incorporation of conductive fillers, like salts, can reduce the necessary actuating voltage. However, several important questions arise about how the type of salt chosen and its concentration will affect not only the activation efficiency of the actuators but also the structure of the hydrogels utilized. In this study, to enhance the electrical conductivity of the hydrogel and lower the necessary activation voltage of the hydrogel actuators, lithium chloride (LiCl) and sodium chloride (NaCl) were incorporated as conductive fillers into the polyvinyl alcohol (PVA) polymer matrix. To determine the deformation of actuators, as well as the activation and relaxation times and efficiencies during activation, linear actuators capable of being activated through extension/contraction (swelling/shrinking) cycles were developed and examined based on the LiCl/NaCl content, applied voltage, and frequency. The main finding is that the required actuating voltage was lowered by up to 20 V by adding an equal mass of salt in relation to the PVA mass content. With a load of around 20 kPa, it was observed that the extension deformation for PVA/NaCl-based actuators can achieve 75%, while in contraction deformation, can reach 17%. Additionally, for the PVA/LiCl-based actuators, the extension deformation can reach 87%, while during contraction deformation, it can reach 22%. The degree of swelling in the PVA/NaCl hydrogels was generally less than that in the PVA/LiCl hydrogels, which was associated with the finding that the actuators prepared from PVA/NaCl hydrogels delivered an output that was 10–15% lower than those made from PVA/LiCl hydrogels across different testing cycles. Furthermore, adding salt increases the degree of crosslinking, which can explain why increased crosslinking leads to reduced deformation when exposed to AC voltage. These actuators can find extensive use in soft robotics, artificial muscles, medical applications, and aerospace industries.

## 1. Introduction

Currently, the quantity of polymer materials created for particular applications by considering their physical and chemical characteristics has significantly risen. These polymeric substances exhibit exceptional qualities, including lightweight, ease of use, quiet operation, biodegradability, rapid response, and satisfactory mechanical characteristics [1,2]. One of the most fascinating types of polymers is referred to as “smart polymers.” Their characteristics can be modified in real time through the use of various external stimuli, including electricity, magnetism, pH, light, and more [3,4]. Their adaptability is remarkable, as they can exist in various shapes and sizes, including solid, liquid, and gel forms, whether nano-, micro-, or macro-scale. Their rapid reaction to internal and external stimuli has resulted in their use across various applications, including fast-responding sensors, controlled drug delivery systems, actuators, and self-healing materials [5,6,7,8]. Electroactive polymers (EAPs) are an important and intriguing category of polymers that demonstrate deformation when exposed to an external electric field. Actuators that utilize EAPs are viewed as a desirable option for electromechanical actuator applications due to their capability to accurately manage the conversion between electrical and mechanical energy, and vice versa, when subjected to an electrical current [3,9]. The widely recognized process for activating ionic EAPs with direct current (DC) power is ion diffusion, leading to changes in the actuator’s dimensions and its shape (bending) as a result of ion mobility in the electrolyte [10]. Ionic EAPs triggered by DC current exhibit multiple disadvantages, such as low strength, slow activation, and challenges in maintaining moisture. They struggle to maintain continuous deformation when utilized with DC power [4,11]. Hydrogels prepared from polyvinyl alcohol (PVA) are a well-known instance of ionic EAPs. PVA is a synthetic polymer that is hydrophilic, biodegradable, and biocompatible [12]. It is extensively employed across numerous sectors, including uses in biomedicine, wastewater management, food packaging, textiles, and paper production [13,14].

**Figure 1:**
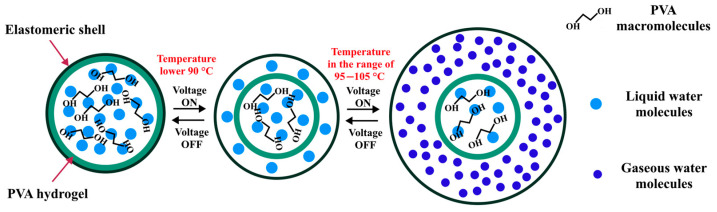
Mechanism diagram of PVA hydrogel activation by AC power.

In our earlier studies [15,16], a new approach for activating polyvinyl alcohol hydrogels as actuators using alternating current (AC) power was proposed. In this method, the actuation mechanism of the actuators is based on the extension and contraction cycles caused by the localized vibrations of ions without any movement toward the electrodes. This process heats the hydrogel, which causes the actuator to expand and transform the water molecules into vapor. In more detail, hydrogel shows a low swelling ratio under ambient conditions due to its hydrogen bond-compressed network structure, and when it is heated after applying AC power, the hydrogen bonds weaken, causing the twisted molecular chains of the polymer to extend. Additionally, when the temperature of the hydrogel surpasses 95 °C, the hydrogen bond-compressed network structure will rupture, causing the water molecules to transition into vapor, resulting in the actuator expanding (Figure 1).

With DC power, the electric field may be partially obstructed due to the migration of electrolyte ions towards the electrodes and their buildup on their surfaces [17,18]. Nevertheless, with AC power, the ion movement associated with the current frequency complicates the observation of the effect, as the variation in the voltage value transferred across the electrolyte can become apparent when the current frequency changes [19,20]. Additionally, in AC power systems, Joule heating is regarded as the primary factor associated with the heating of the electrolyte due to ohmic current. The effect of Joule heating will lead to a change in the density of the electrolyte, the creation of an AC electrothermal flow, and, additionally, the boiling of the electrolyte [21,22]. Moreover, Joule heating can generate AC electro-osmosis flows, significantly influencing the activation process of the hydrogel actuator [23]. In other terms, water moves through the hydrogel, acting as a semi-permeable barrier from the side of the water hydrogel with higher chemical potential to the side with lower chemical potential [24].

In our previous work [15], to reduce the applied AC voltage required to activate PVA hydrogel-based actuators, lithium chloride (LiCl) was incorporated into the hydrogel matrix as a conductive filler, enhancing the electrical conductivity of the PVA hydrogels. It was determined that the required AC voltage for activating the actuator was 20 V. Nevertheless, several crucial questions regarding how the kind of salt (filler) used and its concentration will influence not just the activation efficiency of the actuators but also the structure of the hydrogels employed. In other terms, how the added filler will influence the crosslinking and swelling extent of the PVA hydrogels.

Crosslinking provides hydrogels with a physical framework and elasticity in their network, allowing them to expand or contract. Hydrogels demonstrate considerable volume alterations without altering their structural characteristics, influenced by environmental factors like temperature, pressure, salt content, and pH [25]. The primary mechanisms in activating hydrogel actuators are regarded as swelling and deswelling processes. This sudden change in volume is affected by the chemical characteristics of the polymer chain [26], including the existence of hydrophobic groups, the density of crosslinks, and the interactions between polymer segments, solvents, and salts [27,28].

Hygroscopic hydrogels represent a new group of sorbent materials capable of addressing shortcomings found in other sorbents by offering remarkable water absorption, rapid water capture and release rates, and minimal desorption enthalpies [29,30,31]. Lithium chloride is considered a hygroscopic salt for polymer hydrogels, which therefore enhances water retention capacity and improves the efficiency of heat and moisture exchangers. Additionally, because of its greater hydration energy, LiCl can take in moisture from the environment and sustain the hydrogel’s moisture levels. In reference [32], polyacrylamide hydrogels with LiCl were developed as highly stretchable transparent electrodes for flexible electronics, aiming to improve the water retention capacity of the polyacrylamide hydrogel by including a readily hydratable salt within the hydrogel. The authors demonstrated that the synthesized hydrogels exhibited an enhanced ability to retain water at various levels. Specifically, a polyacrylamide hydrogel containing a high amount of lithium chloride can preserve over 70% of its initial water in conditions with merely 10% relative humidity. In reference [33], investigated the swelling behavior of hydrogels in lithium chloride aqueous solutions, the effects on salt absorption in hydrogels, and the consequent vapor absorption of the created hydrogel–salt composites. They discovered that hydrogels with elevated swelling ratios can absorb more salt solutions while swelling. Consequently, the salt concentration in relation to the polymer will increase, and during absorption, the water vapor intake will more closely resemble that of LiCl. Sodium chloride (NaCl) is anhydrous salt, but it shows hygroscopic properties only because of its impurities. In reference [25], prepared and studied the thermodynamic model for swelling and salt partitioning for the hydrogel of poly(n-isopropyl acrylamide) with the addition of NaCl. They found that the hydration of ions between NaCl and water is the primary reason for the deswelling of hydrogels in aqueous NaCl solutions at elevated NaCl concentrations. Conversely, the intense repulsive forces between NaCl and the polymeric hydrogel are the primary factors influencing salt partitioning. In reference [34], investigated the effect of sodium chloride levels on the physical and oxidative stabilities of filled hydrogel stabilized with heat-denatured whey protein concentrate and high methoxy pectin. They found that as NaCl concentration rose, the thickness of the interfacial layer in filled hydrogels notably increased, while the lightness and whiteness progressively diminished. Additionally, rheological analysis showed that the apparent viscosity and viscoelastic properties diminished progressively with increasing NaCl concentration, primarily attributed to NaCl’s effect on the electrostatic repulsion among droplets, consequently affecting the physical stability of filled hydrogels negatively. In reference [35], they investigated the deformation of PVA hydrogels induced chemically through interaction with diffusing NaCl and KCl solutions. Their findings indicated that PVA hydrogels undergo deformation when salts are present in the pore fluid, and greater deformation occurs with increased salt concentration.

The use of hydrogels, especially those based on PVA, as electroactive-based actuators is considered an urgent task nowadays. Moreover, these hydrogels are available and cheap. In addition, processing them is regarded as simple. However, activating actuators based on hydrogels using AC power is considered a new method that needs to be studied and investigated in detail. In this work, actuators based on PVA hydrogels were prepared by adding NaCl and LiCl as conductive fillers. The influence of salt concentrations on swelling and crosslinking degrees of the hydrogels was investigated. Moreover, the actuating behavior of the prepared actuators based on PVA/salt hydrogels was investigated based on the applied AC voltage and frequencies and salt concentration.

## 2. Results and Discussion

Table 1 and Table 2 present the electrical conductivity of the PVA/salt hydrogels at various frequencies. Here, it should be noted that the values of the electrical conductivity of PVA hydrogel without salt addition were 0.010 ± 0.007 S/m (electrical resistance = 1641 ± 8 Ω) at 50 Hz, and 0.012 ± 0.006 S/m (electrical resistance = 1347 ± 5 Ω) at 500 Hz [16]. It is evident that the introduction of salt and the rise in frequency resulted in an increase in the electrical conductivity values of the PVA/salt hydrogels. This pertains to the idea that increasing the number of ions in the water (solvent) causes these ions to become charged particles capable of conducting an electric current. In addition, Figures S1 and S2 show SEM images of the internal structure of PVA/salt hydrogels. As can be seen, the internal structure of the PVA/salts hydrogels is porous. In Figures S1e,f and S2b, salt particles were visible for PLi4 and PNa4, while no salt particles were observed for the hydrogels of PLi1 and PNa1, which means that all the salt particles were dissolved in the aqueous solvent.

Understanding the molecular arrangement of liquid water, particularly the hydrogen bonds between molecules, is considered very important in hydrogel systems [36]. Hydrogen bonds within the hydrogel network are crucial in the activation process of the actuator when subjected to AC voltage, which means that as the temperature of the hydrogel rises due to the Joule heating effect, the water molecules will convert into a gaseous state following the breakdown of the hydrogen bonds, subsequently causing the volume of the elastomeric shell surrounding the hydrogel to expand, thus activating the actuator. In AC systems, the ohmic current triggers the Joule heating effect, which will then create an AC electrothermal flow, leading to the boiling of water molecules [21,22]. In hydrogel systems, under low frequencies and electric fields, the variation in the dielectric constant of the solution can be minimal [37,38]; while, for highly conductive solutions, temperature variations due to the Joule heating effect will rely on the conductivity and the effective voltage drop across the electrolyte [39]. In this context, variations in the electrolyte’s temperature are associated with alterations in its chemical potential and permittivity [39]. In the presence of an AC electric field, materials with high permittivity exhibit greater polarization than those with low permittivity, indicating a higher energy storage capacity [40]. This implies that incorporating salts like NaCl and LiCl into the hydrogels will reduce their permittivity. Consequently, the impact of Joule heating will occur at a reduced AC voltage, implying that the hydrogel actuators utilizing PVA/salts can be triggered at a lower voltage when compared to those made from PVA.

**Figure 2:**
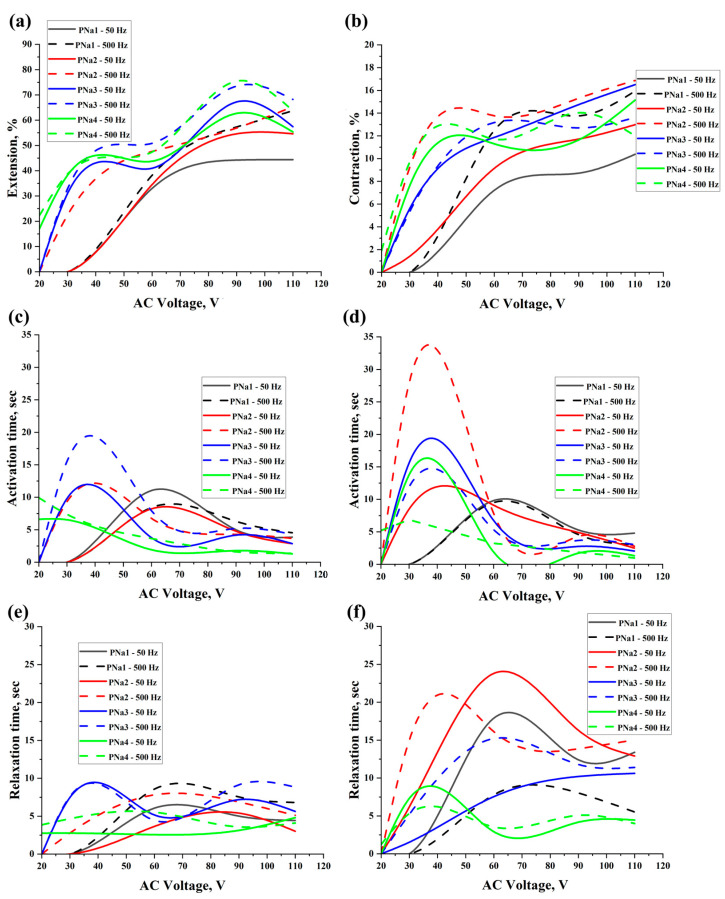
Extension (**a**) and contraction (**b**) values of activated actuators based on PVA/NaCl, activation time of actuators for extension (**c**) and contraction (**d**) deformation, and relaxation time of actuators for extension (**e**) and contraction (**f**) deformation under 50 Hz and 500 Hz.

Figure 2 and Tables S1–S8 show the extension/contraction values, activation and relaxation times for the actuators based on PVA/NaCl hydrogels. As can be seen, the values of the extension deformation were increased by increasing the applied voltage and frequency up to 110 V and 500 Hz, consequently, for PNa1 and PNa2. Whereas, for PNa3 and PNa4, i.e., by increasing NaCl content, the maximum extension deformation was obtained under 90 V and 500 Hz. Moreover, in general, by increasing the NaCl content, the required activation time was decreased; under higher applied voltage, the required activation time was lower. The same behavior was observed for the actuators based on PVA/NaCl under contraction activation. However, it should be noted that for PNa4, i.e., by increasing the NaCl content, the required actuating voltage was decreased up to 20 V. The best results for PNa samples under extension deformation were for PNa4 under 90 V and 500 Hz, which were 75.46 ± 1.96% with activation and relaxation times of 1.61 ± 0.29 and 3.59 ± 0.81 s, respectively, whereas, under contraction deformation, the best results were for PNa2 under 110 V and 500 Hz, which were 16.87 ± 1.17% with activation and relaxation times of 2.77 ± 0.6 and 15.03 ± 4.77 s, respectively.

**Figure 3:**
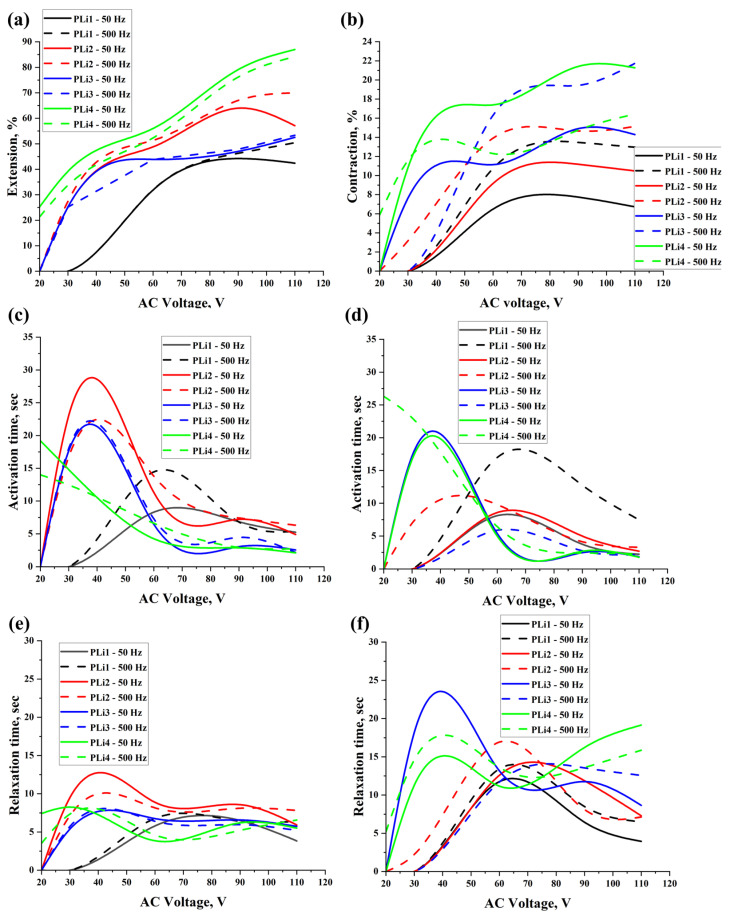
Extension (**a**) and contraction (**b**) values of activated actuators based on PVA/LiCl, activation time of actuators for extension (**c**) and contraction (**d**) deformation, and relaxation time of actuators for extension (**e**) and contraction (**f**) deformation under 50 Hz and 500 Hz.

Figure 3 and Tables S9–S16 show the extension/contraction values, activation, and relaxation times for the actuators based on PVA/LiCl hydrogels. As can be seen, generally, the values of the deformation (extension/contraction) were increased by increasing the applied voltage and frequency. Here, it should be noted that the activation time for PLi samples was higher than the one for the PNa samples. This can be related to the higher hydration energy of LiCl, which means higher adsorption of water, which, in turn, leads to requiring higher energy to breakdown the hydrogen bonds between the water and PVA molecules [32]. The best results for PLi samples under extension deformation were for PLi4 under 110 V and 50 Hz, which were 87 ± 2.3% with activation and relaxation times of 2.1 ± 0.75 and 5.5 ± 1.31 s, respectively, whereas, under contraction deformation, the best results were for PLi3 under 110 V and 500 Hz, which were 21.74 ± 0.95% with activation and relaxation times of 2.17 ± 0.45 and 12.57 ± 3.16 s, respectively. In comparison with our previous work [15], in which a mass of 7 g of PVA was used in the preparation of PVA/LiCl hydrogels, it was found that the deformation values under contraction/extension were higher when lower PVA content was used. Moreover, these deformation values were also higher by increasing the LiCl content. This is related to the amount of formed hydrogen bonds between water and PVA molecules, which means that when the PVA concentration was higher, the amount of hydrogen bonds was higher, which in turn means that the energy required to break down of these bonds will be higher, and the overall values of deformation will be lower.

Moreover, the relaxation time depended on the deformation level of the actuators and increased with greater deformation. Nevertheless, the relaxation time during contraction deformation exceeded that of extension deformation, which is associated with the deformation limit of the woven mesh reaching 20–25%. This indicates that the hydrogel became hotter in the actuators supported by woven mesh to achieve the highest deformation compared to the actuators reinforced by spiral weaves.

Table 3 and Table 4 show the efficiency of PVA/salt actuators. As can be seen in Table 3 and Table 4, and in comparison with our previous works [15,16], the values of efficiency are still low, although after adding salts. Additionally, when compared to our prior study [15], where the PVA content was greater, the efficiency of the actuator was somewhat higher. This indicates that raising the PVA content will reduce the AC power necessary to activate the hydrogel actuators, consequently enhancing the actuator efficiency.

Swelling tests of PVA-based hydrogels provide insights into the material’s structural stability within the polymer matrix [41,42]. The swelling coefficient determines the operational performance of hydrogel-based actuators, where increased swelling rates lead to enhanced deformation capabilities and faster activation and relaxation times when exposed to AC power. Table 5 shows the values of the swelling degree of each hydrogel.

**Figure 4:**
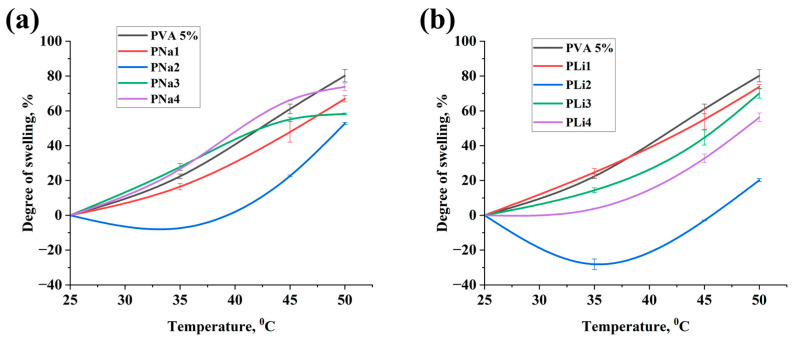
Swelling degree of hydrogels based on (**a**) PVA/NaCl and (**b**) PVA/LiCl.

The minus in the values of the swelling degree indicates that the hydrogel expands when distilled water is added. Therefore, as can be seen in Table 5 and Figure 4, PLi2 had greater potential when used in actuators, as it can save the ability to swell on different temperatures. The deformation data shows that the deformation of the actuator based on PLi2 can reach 70% when a spiral weave was used as an external reinforcement. However, PLi4 had a lower degree of swelling in comparison with PLi2, but it showed better extension deformation values up to 87 ± 2.3% when it was applied to 110 V and 50 Hz. This was due to the concentration of salt in hydrogel. The addition of salt to hydrogel increased the conductivity and reduced the required AC voltage for the activation, that is why the deformation index improved [43,44]. Additionally, as illustrated in Figure 4b, the degree of swelling diminished with higher salt concentrations exceeding 50 wt.% of PVA mass content (PLi3, PLi4, PNa3, and PNa4). This may result from salt influencing the structure of the polymer network and decreasing the space available for water absorption. The presence of salt can modify the interactions between polymer chains and water molecules, resulting in a lower degree of swelling [45,46]. The incorporation of NaCl into the hydrogel matrix demonstrated favorable swelling characteristics, as evidenced by deformation capacities reaching 70% in spiral-woven configurations and 20% in mesh-woven ones. However, the swelling degree of the PVA/NaCl hydrogels was generally lower than the one of the PVA/LiCl hydrogels. This difference in swelling performance was supported by the measurements of deformation of the actuators, which consistently showed that actuators based on PVA/NaCl hydrogels yield their counterparts based on PVA/LiCl hydrogels by 10–15% across multiple testing cycles. The improved actuation characteristics of PVA/LiCl hydrogels can be explained by stronger interactions between Li^+^ ions and water molecules, which collectively promote more efficient water absorption during actuator operation [47].

The degree of crosslinking is a quantitative characteristic of the density of chemical bonds (crosslinking agents) between polymer chains in the hydrogel structure. The degree of crosslinking directly determines the mechanical strength of the hydrogels, forming a denser structure with increasing cross-bond density. However, an increase in the degree of crosslinking in PVA hydrogels limits the mobility of polymer chains, reducing the availability of hydroxyl groups [48,49]. Accordingly, materials with a low degree of crosslinking have a high swelling capacity due to their larger free volume, which positively impacts the operation of the actuator. Table 6 shows the degree of crosslinking of hydrogels.

**Figure 5:**
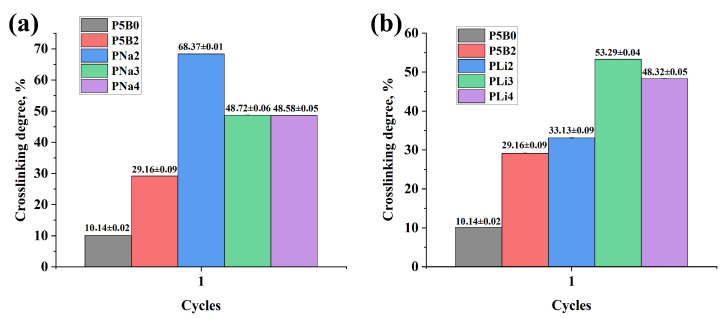
Crosslinking degree of hydrogels based on (**a**) PVA/NaCl and (**b**) PVA/LiCl.

As the hydrogel heats up, this results in a weakening of the hydrogen bonds, which consequently causes the twisted polymer molecular chain to stretch. This will result in a volumetric reduction of the PVA hydrogel along with a decline in its viscosity [50]. In reference [51], polyvinyl alcohol gel was examined as a polymeric gel responsive to temperature changes. They showed that PVA hydrogels possess a crucial transition temperature (with H_2_O) of 37 °C. They indicated that if the hydrogel temperature exceeds 37 °C, the hydrogel can readily release the water molecules, while at temperatures below, the hydrogel can firmly hold onto the water molecules. A high degree of crosslinking indicates the structural stability of the samples. However, during the operation of the actuators, this property negatively affects the swelling of the hydrogel, which leads to low deformation. As can be seen in Table 6 and Figure 5, sample P5B0, which lacks a crosslinking agent, showed the lowest crosslinking degree (10.14 ± 0.02). When sodium tetraborate (borax) was added as a crosslinking agent to a salt-free hydrogel (sample P5B2), the degree of crosslinking was increased significantly to 29.16 ± 0.09%. Also, comparison of the results with control sample P5B0 shows that the addition of salt increases the degree of crosslinking. For example, PNa2, which has the highest crosslinking degree of 68.37 ± 0.01%, exhibited weak deformation under the influence of AC-voltage. In contrast, PLi2, which demonstrated excellent swelling properties, showed only a slight deviation from P5B2 (about 4%) and one of the best deformations, as mentioned earlier. Incorporating LiCl into PVA hydrogels can affect crosslinking and swelling degrees. LiCl may function as a plasticizer, influencing physical crosslinking and possibly changing the swelling characteristics. The extent of crosslinking, either physical or chemical, greatly influences a hydrogel’s ability to absorb fluids and its appropriateness for different uses [52]. As shown in Figure 4b and Figure 5b, when the LiCl concentration exceeds 50 wt.% of the PVA mass content, there is a reduction in the swelling degree of PVA hydrogels and an enhancement in the degree of crosslinking. This is probably due to the denser hydrogel structure created from enhanced crosslinking or interactions [53].

## 3. Conclusions

This study focused on the preparation and analysis of quick-responding linear actuators utilizing PVA with LiCl and NaCl included as conductive fillers. Utilizing AC power aligned with the hydrogel’s water molecules activation mechanism; the molecules converted into a gas due to the Joule heating effect. Two types of reinforcement, a spiral weave, and a woven mesh braided material, were utilized to convert the actuators’ swelling volumetric actuation into a linear form. The deformations of the actuators, including modes of contraction and extension, as well as their efficiencies and the times for activation and relaxation, were examined based on salt concentration, applied voltage, and frequency under an approximate load of 20 kPa. Additionally, the degree of cross-linking and swelling of the prepared hydrogels was examined to assess their effect on the performance of the actuators derived from PVA/salt hydrogels.

It was found that raising the salt concentration led to a reduction in the necessary AC voltage to trigger the actuators due to the enhanced electrical conductivity of the hydrogel. Typically, the values of the deformation of extension/contraction rose with higher applied voltage and frequency. Furthermore, it was discovered that raising the salt concentration reduced the necessary activation time; and at greater applied voltage, the needed activation time was shorter. The key discovery is that the necessary actuating voltage was reduced by as much as 20 V by incorporating an equal mass of salt relative to the PVA mass content. The optimal outcomes for PNa samples during extension deformation were observed in PNa4 at 90 V and 500 Hz, yielding 75.46 ± 1.96% with activation and relaxation durations of 1.61 ± 0.29 and 3.59 ± 0.81 s, respectively; in contrast, for contraction deformation, the best performance was noted in PNa2 at 110 V and 500 Hz, yielding 16.87 ± 1.17% with activation and relaxation times of 2.77 ± 0.6 and 15.03 ± 4.77 s, respectively. Furthermore, the optimal outcomes for PLi samples during extension deformation were observed for PLi4 at 110 V and 50 Hz, yielding 87 ± 2.3% with activation and relaxation durations of 2.1 ± 0.75 and 5.5 ± 1.31 s, respectively; conversely, during contraction deformation, the best results were recorded for PLi3 at 110 V and 500 Hz, resulting in 21.74 ± 0.95% with activation and relaxation times of 2.17 ± 0.45 and 12.57 ± 3.16 s, respectively.

According to the swelling degree values of the prepared hydrogels, it was observed that the swelling degree of the PVA/NaCl hydrogels was typically lower than that of the PVA/LiCl hydrogels, which was linked to the observation that the actuators made from PVA/NaCl hydrogels produced a lower output compared to those made from PVA/LiCl hydrogels by 10–15% over various testing cycles. This may be connected to the enhanced interactions between Li+ ions and water molecules, which together foster improved water absorption during the operation of the actuator. In addition, and according to the crosslinking degree values of the prepared hydrogels, it was found that the inclusion of salt enhances the level of crosslinking. This can clarify why a greater degree of crosslinking results in less deformation when subjected to AC voltage and vice versa.

These PVA/salt hydrogel-based actuators have numerous applications in the fields of soft robotics, artificial muscles, medicine, and aerospace.

## 4. Materials and Methods

### 4.1. Materials

Polyvinyl alcohol (PVA) acquired from “Ruskhim Ltd.” (Moscow, Russia) was used, and it has a typical molecular weight of 105,000 g/mol, with a hydrolysis degree of 99%. Sodium tetraborate (borax), with a molecular weight of 381.37 g/mol, served as a crosslinking agent. Distilled water served as the solvent for preparing the PVA hydrogel. Anhydrous lithium chloride (LiCl) with a molecular weight of 42.39 g/mol and sodium chloride (NaCl) with a molecular weight of 58.44 g/mol in pure form was utilized as conductive fillers. To prepare actuators based on PVA/salts hydrogels: latex balloons used as an elastomeric shell; an external reinforcement composed of 0.5 mm diameter polycaprolactam fiber in a spiral weave with an internal diameter of 7 mm; an external reinforcement made of woven mesh polyethylene terephthalate with an internal diameter of 8 mm; conductive copper wires acting as electrodes; heat-shrinkable tubes crafted from polyolefins, measuring 10 mm long with diameters of 4 mm and 2 mm. Heat-shrinkable tubes are necessary for correctly linking and securing the external reinforcements, elastomeric casing, and electrodes in the prepared actuators.

### 4.2. PVA Hydrogels Preparation

**Figure 6:**
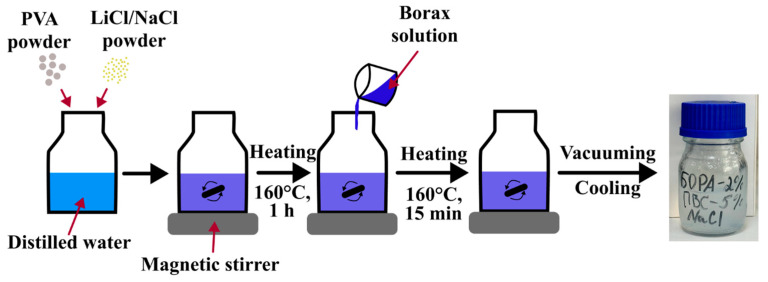
Schematic process of preparation of PVA hydrogel.

Initially, the PVA/salt mixture in distilled water was heated at 160 °C for 1 h with the aid of a magnetic stirrer MS7-H550-S (Scilogex, Cromwell, CT, USA). Next, a borax solution in distilled water was incorporated and mixed at the same temperature for 15 min (Figure 6). Subsequently, the air bubbles from the PVA/salt/borax solutions were transferred through vacuuming using a H300*d623 vacuum chamber with ZSN 3S pump (Zhejiang Zhensheng Mechanical & Electrical Technology Co., Ltd., Wenling, Zhejiang, China). Ultimately, the prepared solutions were stored in the refrigerator at 4 °C for later use.

A total of 100 mL of distilled water was utilized to produce all PVA hydrogels using 5 g of PVA (0.00048 mol/L) and 0.1 g of borax (0.0000026 mol/L). Hydrogels with varying salt concentrations were created to examine how these levels affect the deformation of the actuator as well as its activation and relaxation durations. Table 7 shows the salt content in the hydrogels that were prepared. The used concentrations of salt were chosen based on the mass content of used PVA. In other words, concentrations of 10, 30, 50, and 100 wt. of the polymer mass in hydrogel were used and studied in this work.

### 4.3. Hydrogel Actuators Preparation

**Figure 7:**
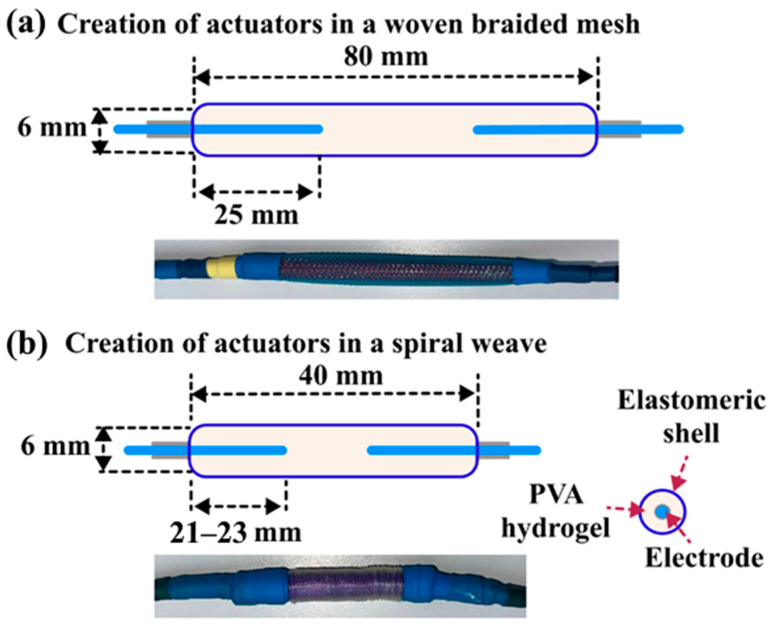
Schematic representation of the hydrogel actuators along with (**a**) woven mesh and (**b**) spiral weave external reinforcements.

The process for preparing the hydrogel actuators was thoroughly detailed in our earlier publication [15]. In summary, 4 mL of hydrogel was injected into a flexible elastomeric casing measuring 40–80 ± 5 mm in length and 6 ± 0.5 mm in inner diameter with a syringe. The shell’s two ends were sealed and attached to the outer reinforcement and copper electrodes with heat-shrinkable tubing. Figure 7 shows a diagram of the hydrogel actuators accompanied by two types of external support. The inner diameters of the two external reinforcements measured 7 ± 0.5 mm.

### 4.4. Methods

#### 4.4.1. Electrical Conductivity Assessment of Hydrogel Actuators

The electrical conductivity of actuators prepared from PVA hydrogels was evaluated using a four-point probe resistivity measurement system (RLC meter AKIP-6112/2, AKIP™, Moscow, Russia), which can measure electrical capacitance ranging from 0.01 nF to 10 F. At a voltage of 2 V, a minimum of three measurements for each hydrogel composite were performed with two frequency values of 50 and 500 Hz.

#### 4.4.2. Actuation Tests

To study the actuation deformation, activation, and relaxation times, actuators with different concentrations of LiCl and NaCl were subjected to AC voltage levels ranging from 20 to 110 V and two frequency settings of 50 and 500 Hz while under a load of 50 g (Figure S3). The frequencies of 50 Hz and 500 Hz were applied as two control points (50 Hz as a similar value to general electrical supplement and 500 Hz as a high value of frequency of 10 times). An AC power source (MATRIX APS-7100 AC Power Source, MATRIX TECHNOLOGY INC., Shenzhen, China) was employed to generate AC voltage, offering an operating voltage range of 0 to 310 V and a frequency range of 45 to 500 Hz.

#### 4.4.3. Actuation Deformation Assessment

**Figure 8:**
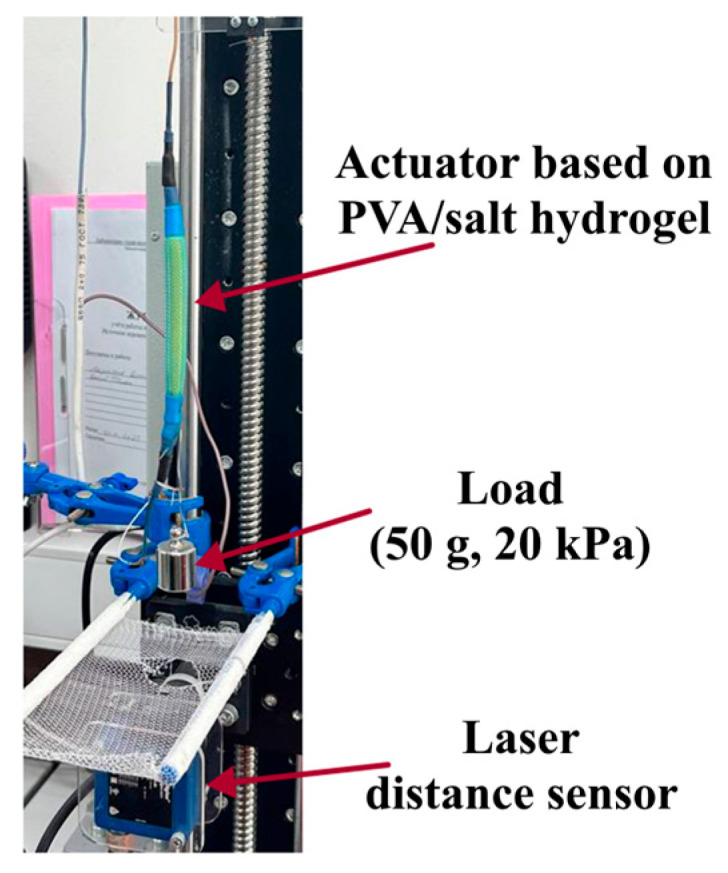
Measurement of actuation deformation by laser detector.

The deformation values for contraction and extension of the hydrogel actuators were measured with a high-performance laser distance sensor, the Wenglor YP11MGVL80 (Wenglor sensoric GmbH, Tettnang, Germany), which features a linearity of 0.5%, a measuring range of 50 mm, and a resolution of 20 µm, as illustrated in Figure 8. Information on deformation values, activation, and relaxation times was collected and evaluated through a USB interface (LA-2USB-14) (LLC Rudnev-Shilyaev, Moscow, Russia) with PowerGraph software version 3.3.11 (DISoft, Moscow, Russia). The activation time was defined as the period from the application of voltage to the maximum peak of deformation, whereas the relaxation time was recorded from when the voltage was switched off to the initial displacement level. Three actuators of each prepared hydrogel were tested to assess the actuation deformation.

The actuator efficiency was calculated in the following manner:

ƞ = P_2_/P_1_ × 100 (1)

where ƞ is the efficiency (%), P_1_ is the supplied electrical power (watt), and P_2_ is the useful mechanical power generated by the actuator (watt). P_1_ and P_2_ were calculated as follows:

P_1_ = V·I (2)

P_2_ = m·a·L/t (3)

where V is the voltage (volts), I is the current (Amps), m is the applied load (kg), a is the acceleration (9.81 m/s^2^), L is the displacement (m), and t is the activation time (sec).

It should be noted that the effective mechanical power produced by the hydrogel actuator was assessed for a length of 1 m of actuator.

#### 4.4.4. Measurement of Swelling of PVA/Salt Hydrogel

The swelling rates of 4 g of PVA/salts hydrogel in distilled water were examined by measuring the increase in water content in gel samples shaped in glass cups and submerged in 50 mL of distilled water [42]. Analytical electronic scales APTP 456 series (Shenzhen Amput Electronic Technology Co.,Ltd, Shenzhen, Guangdong, China) were used to weigh the PVA hydrogel samples. At least 3 PVA hydrogel samples were measured for each PVA/salt hydrogel. Swelling rates were observed at 35, 45, and 50 °C. The extent of swelling was determined using the equation below:

(4) $$Swelling\;degree\left(\%\right)=\frac{W_{i}-W_{s}}{W_{i}}.100$$

where W_i_ represents the initial weight of the obtained PVA/salt hydrogel, while W_s_ denotes the swollen weight of the same PVA hydrogel after being immersed for time (t) in distilled water.

#### 4.4.5. Investigation of Crosslinking Degree of PVA/Salt Hydrogels

Variations in the weight alterations of the dried PVA gels under rinsed and unrinsed conditions for the same hydrogel volume can be considered an indicator of the crosslinking extent [42]. The calculation of the index was done as follows:

(5) $$Crosslinking\;degree\left(\%\right)=\frac{W_{i}-W_{f}}{W_{i}}.100$$

where W_i_ and W_f_, are the weights of the dried PVA hydrogel before and after rinsing and extraction.

Four grams of PVA hydrogels were measured and then dehydrated under vacuum at 90 °C until the dry mass reached a stable weight (w_i_). Almost the same weights of different samples of the identical PVA hydrogels were measured and subsequently soaked in 100 mL of distilled water for 24 h. Afterward, the gel block was removed from the rinse water, dried at 90 °C in a vacuum until all moisture was eliminated, and then weighed (w_f_). At least 3 hydrogel samples were measured for each prepared hydrogel.

## Figures and Tables

**Table 1:** Electrical conductivity of PVA/LiCl hydrogels by the four-probe method.

Material	PLi1		PLi2		PLi3		PLi4	
Frequency, Hz	50	500	50	500	50	500	50	500
Electrical conductivity, S/m	0.016 ± 0.003	0.024 ± 0.002	0.049 ± 0.006	0.064 ± 0.003	0.071 ± 0.002	0.092 ± 0.001	0.094 ± 0.002	0.122 ± 0.002

**Table 2:** Electrical conductivity of PVA/NaCl hydrogels by the four-probe method.

Material	PNa1		PNa2		PNa3		PNa4	
Frequency, Hz	50	500	50	500	50	500	50	500
Electrical conductivity, S/m	0.022 ± 0.004	0.025 ± 0.002	0.033 ± 0.002	0.036 ± 0.003	0.088 ± 0.001	0.093 ± 0.003	0.208 ± 0.004	0.222 ± 0.002

**Table 3:** The efficiency of the actuator based on PVA hydrogel reinforced with spiral weave (extension deformation) under conditions of 500 Hz.

Material Code (Activation Voltage)	PNa1 (110 V)	PNa2 (110 V)	PNa3 (90 V)	PNa4 (90 V)	PLi1 (110 V)	PLi2 (110 V)	PLi3 (110 V)	PLi4 (110 V)
Current, A	0.031 ± 0.003	0.046 ± 0.004	0.069 ± 0.004	0.055 ± 0.005	0.032 ± 0.001	0.033 ± 0.002	0.027 ± 0.003	0.088 ± 0.006
Efficiency, %	2.02 ± 0. 12	1.74 ± 0. 31	1.11 ± 0.11	4.60 ± 0.21	1.32 ± 0.12	1.55 ± 0.22	4.11 ± 0.33	1.60 ± 0.01

**Table 4:** The efficiency of the actuator based on PVA hydrogel reinforced with fabric woven mesh (contraction deformation) under 500 Hz.

Material Code (Activation Voltage)	PNa1 (110 V)	PNa2 (110 V)	PNa3 (110 V)	PNa4 (110 V)	PLi1 (110 V)	PLi2 (110 V)	PLi3 (110 V)	PLi4 (110 V)
Current, A	0.031 ± 0.002	0.057 ± 0.003	0.067 ± 0.003	0.075 ± 0.005	0.043 ± 0.001	0.062 ± 0.003	0.069 ± 0.002	0.045 ± 0.004
Efficiency, %	0.81 ± 0.01	0.50 ± 0.02	0. 31 ± 0.01	0.71 ± 0.02	0.22 ± 0.01	0. 38 ± 0.01	0.67 ± 0.03	0.83 ± 0.01

**Table 5:** Swelling degree (SD) of each hydrogel.

Material Code	SD at 35 °C, %	SD at 45 °C, %	SD at 50 °C, %
PNa1	16.48 ± 1.79	47.88 ± 5.87	66.98 ± 1.84
PNa2	−7.38 ± 0.23	22.65 ± 0.44	52.75 ± 0.59
PNa3	27.80 ± 1.91	55.08 ± 1.25	58.20 ± 0.49
PNa4	26.60 ± 0.98	66.18 ± 0.30	73.80 ± 2.18
PLi1	24.68 ± 2.22	55.13 ± 5.81	73.80 ± 1.21
PLi2	−28.10 ± 3.01	−2.95 ± 0.25	20.18 ± 0.86
PLi3	14.38 ± 1.42	44.63 ± 4.34	70.05 ± 2.82
PLi4	3.78 ± 0.25	32.68 ± 2.46	56.28 ± 2.42
P5B2 *	22.38 ± 1.29	61.08 ± 2.78	80.20 ± 3.56

* P5B2 (PVA—5 g, borax—0.1 g).

**Table 6:** Degree of crosslinking of hydrogels.

Material Code	PNa2	PNa3	PNa4	PLi2	PLi3	PLi4	P5B0 *	P5B2 *
Crosslinking degree, %	68.37 ± 0.01	48.72 ± 0.06	48.58 ± 0.05	33.13 ± 0.09	53.29 ± 0.04	48.32 ± 0.05	10.14 ± 0.02	29.16 ± 0.09

* P5B0 (PVA—5 g, borax—0 g), P5B2 (PVA—5 g, borax—0.1 g).

**Table 7:** The contents of salts in the hydrogels that were prepared.

**Material No.**	**Material Code**	**LiCl Content, g**	**LiCl Concentration, mol/L**	**NaCl Content, g**	**NaCl Concentration, mol/L**
1	PLi1	0.5	0.1180	-	-
2	PLi2	1.5	0.3539	-	-
3	PLi3	2.5	0.5898	-	-
4	PLi4	5.0	1.1797	-	-
5	PNa1	-	-	0.5	0.0856
6	PNa2	-	-	1.5	0.2567
7	PNa3	-	-	2.5	0.4278
8	PNa4	-	-	5.0	0.8558

## Data Availability

Data are available from the Russian Science Foundation (RSF), https://rscf.ru/en/project/24-23-00558/.

## Supplementary Materials

The following supporting information can be downloaded at: https://www.mdpi.com/article/10.3390/gels11070484/s1, Figure S1. SEM images of the internal structure of PVA/LiCl hydrogels: (a,b) PVA, (c,d) PLi1, (d,f) PLi4; Figure S2. SEM images of the internal structure of PVA/NaCl hydrogels: (a) PNa1, (b) PNa4; Figure S3. Working mechanism of linear actuators based on PVA/salt hydrogel reinforced by (a) spiral weave and (b) woven mesh braided; Figure S4: Contraction values of activated actuators based on PVA/NaCl under (a) 50 Hz and (b) 500 Hz; Figure S5: Extension values of activated actuators based on PVA/NaCl under (a) 50 Hz and (b) 500 Hz. Table S1: Extension, activation, and relaxation values for hydrogel actuators of PNa1; Table S2. Extension, activation, and relaxation values for hydrogel actuators of PNa2; Table S3: Extension, activation, and relaxation values for hydrogel actuators of PNa3; Table S4: Extension, activation, and relaxation values for hydrogel actuators of PNa4; Table S5: Contraction, activation, and relaxation values for hydrogel actuators of PNa1; Table S6: Contraction, activation, and relaxation values for hydrogel actuators of PNa2; Table S7: Contraction, activation, and relaxation values for hydrogel actuators of PNa3; Table S8: Contraction, activation, and relaxation values for hydrogel actuators of PNa4; Table S9: Extension, activation, and relaxation values for hydrogel actuators of PLi1; Table S10. Extension, activation,, and relaxation values for hydrogel actuators of PLi2; Table S11: Extension, activation and relaxation values for hydrogel actuators of PLi3; Table S12: Extension, activation, and relaxation values for hydrogel actuators of PLi4; Table S13: Contraction, activation, and relaxation values for hydrogel actuators of PLi1; Table S14: Contraction, activation, and relaxation values for hydrogel actuators of PLi2; Table S15: Contraction, activation and relaxation values for hydrogel actuators of PLi3; Table S16: Contraction, activation, and relaxation values for hydrogel actuators of PLi4.

## Abbreviations

The following abbreviations are used in this manuscript:

PVA	polyvinyl alcohol
LiCl	lithium chloride
NaCl	sodium chloride
EAPs	electroactive polymers
DC	direct current
AC	alternating current
