# Supplementary material for: The Influence of Concentration and Type of Salts on the Behaviour of Linear Actuators Based on PVA Hydrogel Activated by AC Power
Source: Gels. 2025 Jun 23;11(7):484. doi: 10.3390/gels11070484 (PMC12294192; doi:10.3390/gels11070484)
Supplement: Supplementary file 1 [file gels-11-00484-s001.zip › gels-3697845-supplementary.pdf]

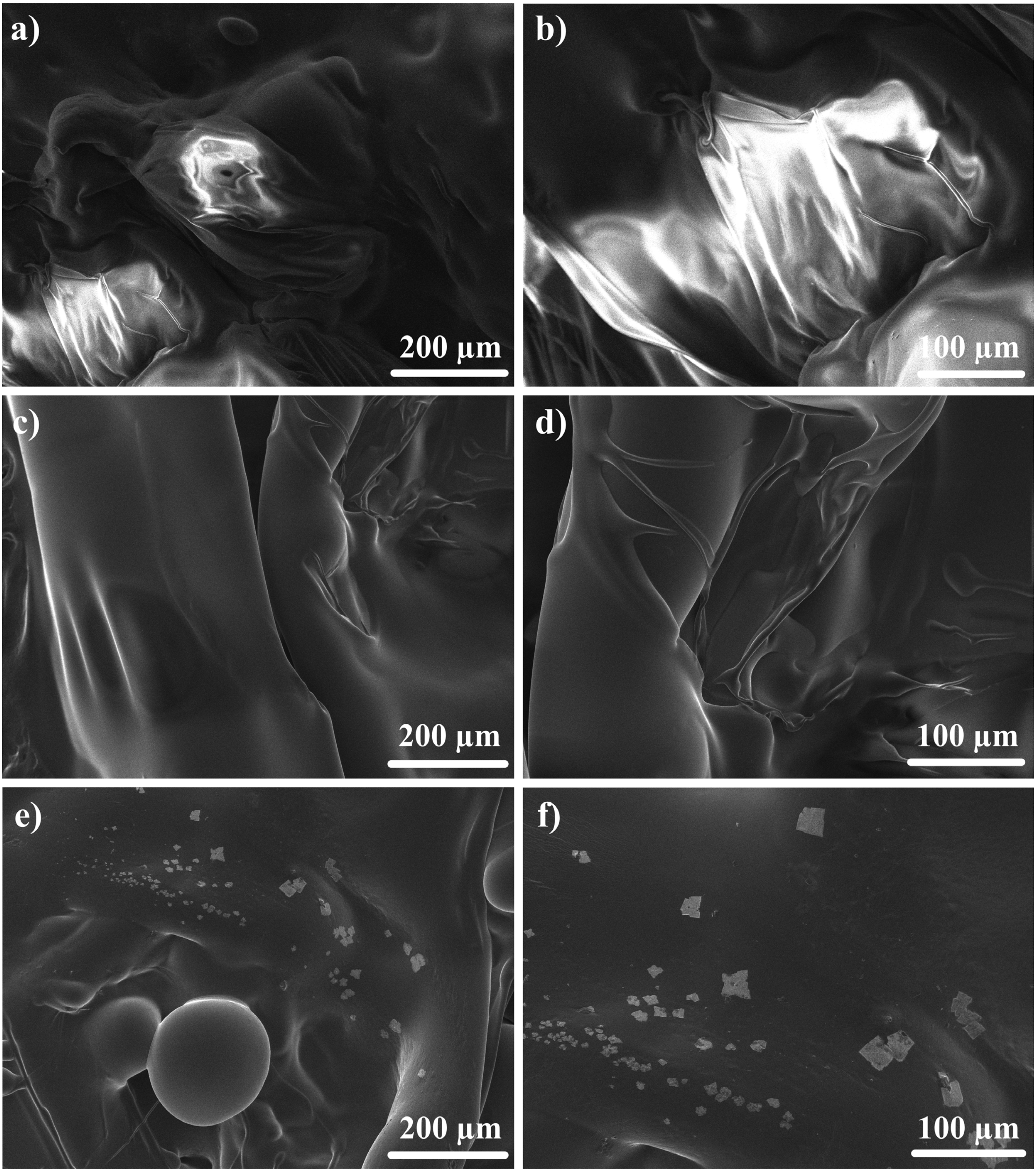

Figure S1. SEM images of the internal structure of PVA/LiCl hydrogels: (a, b) PVA, (c, d) PLi1, (d, f) PLi4.

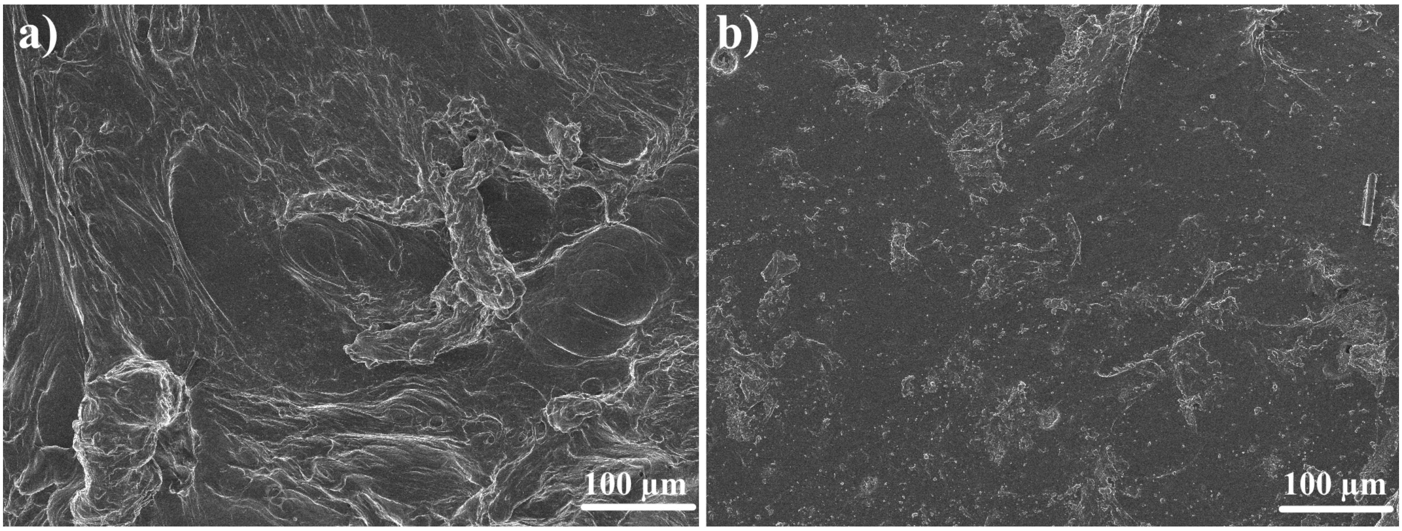

Figure S2. SEM images of the internal structure of PVA/NaCl hydrogels: (a) PNa1, (b) PNa4.

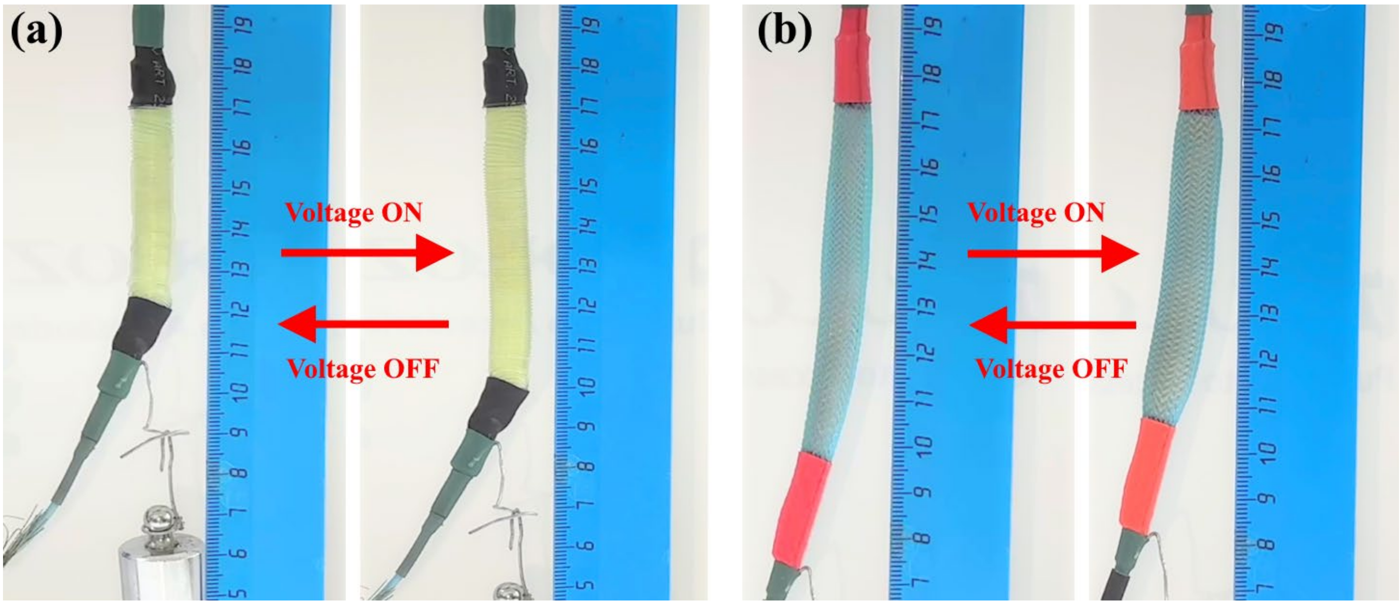

Figure S3. Working mechanism of linear actuators based on PVA/salt hydrogel reinforced by (a) spiral weave and (b) woven mesh braided.

Table S1. Extension, activation and relaxation values for hydrogel actuators of PNa1.

Voltage, V	20		30		60		90		110	
Frequency, Hz	50	500	50	500	50	500	50	500	50	500
Deformation, %	Low voltage	Low voltage	Low voltage	Low voltage	33.01 ±1.10	38.17± 0.10	44.20±1.20	57.32±0.73	44.40±2.20	63.73±0.60
Activation time, sec	-	-	-	-	11.03 ±0.65	8.42±0.29	4.99±0.57	6.30±0.20	3.86±0.80	4.54±0.16
Relaxation time, sec	-	-	-	-	6.06± 0.41	8.57±0.07	5.01±0.52	7.54±0.49	4.43±0.37	6.81±0.22

Table S2. Extension, activation and relaxation values for hydrogel actuators of PNa2.

Voltage, V	20		30		60		90		110	
Frequency, Hz	50	500	50	500	50	500	50	500	50	500
Deformation, %	Low voltage	Low voltage	Low voltage	22.52 ±0.23	34.51 ±0.52	47.63± 1.08	54.59±0.75	56.93±0.44	54.62±0.78	65.32±0.62
Activation time, sec	-	-	-	9.32± 0.13	8.22± 0.15	6.75±0.03	4.77±0.48	4.29±0.12	2.86±0.12	3.67±0.13
Relaxation time, sec	-	-	-	2.78± 0.07	3.65± 0.20	7.82±0.10	5.40±0.45	6.87±0.08	3.01±0.29	5.12±0.13

Table S3. Extension, activation and relaxation values for hydrogel actuators of PNa3.

Voltage, V	20		30		60		90		110	
Frequency, Hz	50	500	50	500	50	500	50	500	50	500
Deformation, %	Low voltage	Low voltage	31.07 ±1.60	32.9± 0.40	40.98 ±0.38	50.85± 0.45	67.24±2.67	73.51±0.21	57.4±2.23	68.21±1.11
Activation time, sec	-	-	9.81± 0.11	15.52 ±0.13	3.78± 0.11	7.93±0.11	4.17±0.13	5.19±0.34	2.86±0.33	4.30±0.52
Relaxation time, sec	-	-	7.47± 0.34	7.61± 0.49	5.16± 0.59	4.45±0.49	7.18±0.35	9.14±0.92	5.63±0.15	8.82±0.82

Table S4. Extension, activation and relaxation values for hydrogel actuators of PNa4.

Voltage, V	20		30		60		90		110	
Frequency, Hz	50	500	50	500	50	500	50	500	50	500
Deformation, %	17.10 ±8.60	22.30 ±1.01	38.27 ±0.45	38.70 ±2.60	43.74 ±0.33	47.43± 2.38	62.70±0.67	75.46±1.96	55.34±1.45	63.72±1.98
Activation time, sec	6.62± 3.37	9.93± 0.43	6.54± 0.18	7.38± 0.92	1.93± 0.01	3.65±0.75	1.78±0.03	1.61±0.29	1.29±0.02	1.37±0.36
Relaxation time, sec	2.76± 1.13	3.86± 0.28	2.77± 0.44	4.67± 0.57	2.56± 0.19	5.53±0.47	3.13±0.05	3.59±0.81	4.81±0.49	4.10±0.18

Table S5. Contraction, activation and relaxation values for hydrogel actuators of PNa1.

Voltage, V	20		30		60		90		110	
Frequency, Hz	50	500	50	500	50	500	50	500	50	500
Deformation, %	Low voltage	Low voltage	Low voltage	Low voltage	7.21± 1.54	12.48± 1.32	8.72±0.79	13.76±2.61	10.38±0.87	15.94±1.76
Activation time, sec	-	-	-	-	9.80± 0.65	9.50±4.447	5.30±1.10	4.60±1.94	4.80±0.54	3.02±0.38
Relaxation time, sec	-	-	-	-	17.90 ±3.75	7.79±1.73	12.40±2.50	8.03±4.55	13.40±1.45	5.52±1.17

Table S6. Contraction, activation and relaxation values for hydrogel actuators of PNa2.

Voltage, V	20		30		60		90		110	
Frequency, Hz	50	500	50	500	50	500	50	500	50	500
Deformation, %	Low voltage	Low voltage	1.41± 1.32	9.24± 1.61	9.14± 0.48	13.77± 2.13	11.72±0.95	15.31±2.37	12.98±1.73	16.87±1.17
Activation time, sec	-	-	8.50± 2.98	27.88 ±4.88	9.10± 1.32	7.84±1.69	4.90±0.76	4.28±0.05	2.50±0.23	2.77±0.60
Relaxation time, sec	-	-	6.2±5.65	15.03 ±8.61	23.8± 1.69	16.12± 6.62	16.3±1.84	13.86±0.46	12.9±3.47	15.03±4.77

Table S7. Contraction, activation and relaxation values for hydrogel actuators of PNa3.

Voltage, V	20		30		60		90		110	
Frequency, Hz	50	500	50	500	50	500	50	500	50	500
Deformation, %	Low voltage	Low voltage	5.60±2.76	5.34±4.29	11.86±1.16	13.17±1.07	14.75±4.25	12.70±0.60	16.50±2.90	13.63±1.60
Activation time, sec	-	-	15.60±5.30	11.90±6.9	6.75±1.15	5.34±2.04	2.75±0.95	3.66±0.84	2.04±0.62	3.03±1.87
Relaxation time, sec	-	-	1.54±3.98	5.21±6.84	7.55±2.70	15.21±7.79	10.20±2.60	11.79±8.04	10.62±4.08	11.40±2.60

Table S8. Contraction, activation and relaxation values for hydrogel actuators of PNa4.

Voltage, V	20		30		60		90		110	
Frequency, Hz	50	500	50	500	50	500	50	500	50	500
Deformation, %	Low voltage	1.90±3.80	7.62±4.60	9.71±1.71	11.30±0.10	11.71±1.71	11.65±1.25	14.03±2.37	15.15±0.17	12.02±0.50
Activation time, sec	-	5.20±0.70	13.78±4.38	6.85±3.57	1.90±0.29	3.24±0.16	1.70±0.58	1.86±1.32	1.32±0.41	0.98±1.32
Relaxation time, sec	-	1.24±0.49	7.32±3.2	5.31±2.71	2.92±1.93	3.54±1.49	4.18±2.42	5.07±0.90	4.46±0.12	4.04±1.34

Table S9. Extension, activation and relaxation values for hydrogel actuators of PLi1.

Voltage, V	20		30		60		90		110	
Frequency, Hz	50	500	50	500	50	500	50	500	50	500
Deformation, %	Low voltage	Low voltage	Low voltage	Low voltage	31.87±1.56	31.95±1.85	44.20±1.30	46.28±4.42	42.40±3.92	50.43±4.67
Activation time, sec	-	-	-	-	8.30±1.82	14.45±0.98	6.83±2.30	6.80±0.55	5.24±0.18	5.38±0.88
Relaxation time, sec	-	-	-	-	5.79±0.31	6.77±0.23	6.5±01.30	6.40±2.15	3.83±1.48	6.40±2.15

Table S10. Extension, activation and relaxation values for hydrogel actuators of PLi2.

Voltage, V	20		30		60		90		110	
Frequency, Hz	50	500	50	500	50	500	50	500	50	500
Deformation, %	Low voltage	Low voltage	25.21±1.49	27.48±2.98	48.8±4.90	51.33±2.33	63.97±5.40	67.03±9.22	57.10±1.80	70.14±6.30
Activation time, sec	-	-	23.06±4.73	16.76±0.64	11.31±1.01	13.80±1.40	7.21±0.36	7.42±0.88	4.89±1.49	6.31±0.69
Relaxation time, sec	-	-	9.50±1.18	7.14±0.56	8.88±0.95	8.16±1.14	8.57±1.09	8.11±0.71	5.92±0.58	7.82±0.48

Table S11. Extension, activation and relaxation values for hydrogel actuators of PLi3.

Voltage, V	20		30		60		90		110	
Frequency, Hz	50	500	50	500	50	500	50	500	50	500
Deformation, %	Low voltage	Low voltage	25.20±1.10	25.18±0.58	43.88±2.62	43.80±5.41	47.02±1.72	47.79±3.01	52.53±2.42	53.36±5.76
Activation time, sec	-	-	17.68±1.62	18.02±0.2	6.45±1.22	7.29±1.19	3.04±1.29	4.45±1.05	2.54±0.66	2.14±0.72
Relaxation time, sec	-	-	5.38±1.42	5.72±0.57	6.75±1.05	6.41±1.66	6.57±1.24	5.96±0.82	5.74±0.54	5.21±1.19

Table S12. Extension, activation and relaxation values for hydrogel actuators of PLi4.

Voltage, V	20		30		60		90		110	
Frequency, Hz	50	500	50	500	50	500	50	500	50	500
Deformation, %	25.2±4.50	21.34±1.09	39.2±3.60	33.73±1.47	56.14±3.20	52.30±0.93	79.09±4.81	76.17±6.13	87.04±2.30	84.20±4.90
Activation time, sec	19.2±0.8	14±1.01	14.81±3.85	12.46±1.14	4.25±0.55	6.62±0.12	2.86±0.64	2.94±0.19	2.10±0.75	2.71±0.31
Relaxation time, sec	7.42±2.18	3.52±0.93	8.25±1.45	7.34±0.73	3.85±1.05	4.60±0.10	6.20±1.73	5.21±0.19	5.50±1.31	6.56±1.08

Table S13. Contraction, activation and relaxation values for hydrogel actuators of PLi1.

Voltage, V	20		30		60		90		110	
Frequency, Hz	50	500	50	500	50	500	50	500	50	500
Deformation, %	Low voltage	Low voltage	Low voltage	Low voltage	6.49±0.88	10.74±0.44	7.74±0.93	13.47±0.9	6.72±1.2	12.96±0.26
Activation time, sec	-	-	-	-	8.10±1.82	16.98±3.98	3.74±1.41	12.75±3.15	2.22±0.74	7.48±2.72
Relaxation time, sec	-	-	-	-	11.75±3.20	13.42±1.08	6.44±1.27	8.50±0.13	3.94±0.89	6.49±2.46

Table S14. Contraction, activation and relaxation values for hydrogel actuators of PLi2.

Voltage, V	20		30		60		90		110	
Frequency, Hz	50	500	50	500	50	500	50	500	50	500
Deformation, %	Low voltage	Low voltage	Low voltage	3.17±2.70	9.17±1.04	13.91±1.98	11.18±0.04	14.66±2.11	10.48±1.75	15.14±2.47
Activation time, sec	-	-	-	6.95±2.30	8.58±0.28	9.79±1.41	4.99±0.91	4.13±0.37	2.68±0.45	3.29±1.36
Relaxation time, sec	-	-	-	2.23±0.44	12.59±0.45	16.93±5.41	11.87±0.63	8.10±3.25	7.28±1.73	7.13±3.39

Table S15. Contraction, activation and relaxation values for hydrogel actuators of PLi3.

Voltage, V	20		30		60		90		110	
Frequency, Hz	50	500	50	500	50	500	50	500	50	500
Deformation, %	Low voltage	Low voltage	7.63±0.96	Low voltage	11.14±1.10	16.3±0.77	14.90±3.60	19.43±1.43	14.28±3.29	21.74±0.95
Activation time, sec	-	-	17.22±4.84	-	5.40±3.05	5.88±0.55	2.47±0.71	2.74±0.51	1.83±0.40	2.17±0.45
Relaxation time, sec	-	-	18.20±1.02	-	13.17±3.61	11.71±0.92	11.76±3.45	13.55±4.95	8.65±1.65	12.57±3.16

Table S16. Contraction, activation and relaxation values for hydrogel actuators of PLi4.

Voltage, V	20		30		60		90		110	
Frequency, Hz	50	500	50	500	50	500	50	500	50	500
Deformation, %	Low voltage	5.87±1.45	10.82±0.52	11.59±1.48	17.39±0.92	12.30±1.10	21.41±0.91	14.75±1.63	21.27±0.88	16.43±1.47
Activation time, sec	-	26.30±7.51	16.70±6.26	23.04±1.03	4.99±0.34	6.44±0.90	2.67±0.43	2.41±0.49	1.75±0.65	2.26±0.19
Relaxation time, sec	-	5.19±1.91	11.29±0.82	14.61±1.19	11.16±0.54	13.60±2.50	16.22±4.91	13.60±3.10	19.15±1.85	15.87±1.57

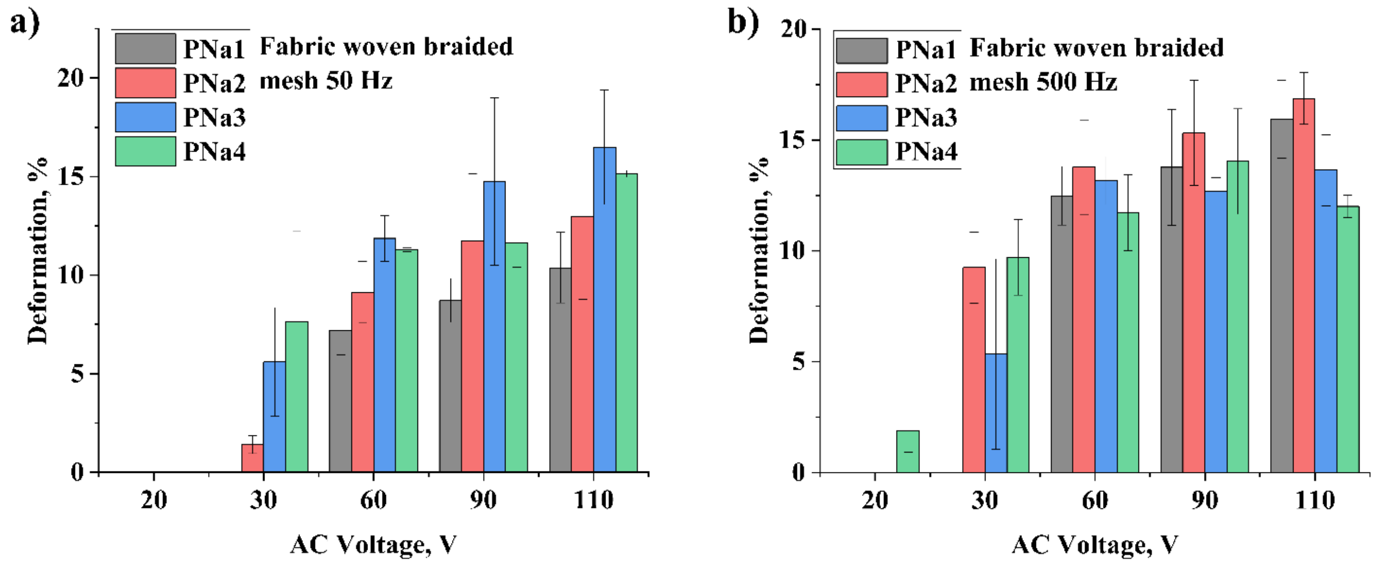

Figure S4. Contraction values of activated actuators based on PVA/NaCl under (a) 50 Hz and (b) 500 Hz.

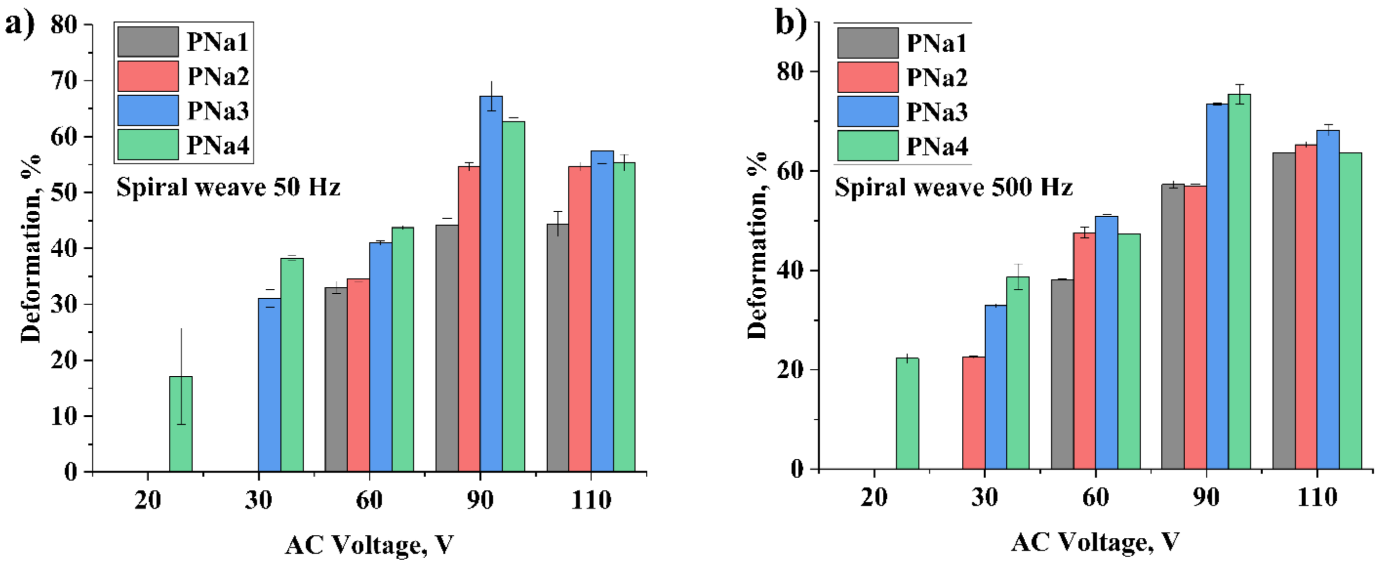

Figure S5. Extension values of activated actuators based on PVA/NaCl under (a) 50 Hz and (b) 500 Hz.
